# Currencies of recognition: What rewards and recognition do Canadian distributed medical education preceptors value?

**DOI:** 10.12688/mep.17540.1

**Published:** 2022-03-01

**Authors:** Aaron Johnston, Rebecca Malhi, Nicholas Cofie, Ruzica Jokic, James Goertzen, Tom Smith-Windsor, Edward Makwarimba, Marie-Hélène Girouard, Sandra Badcock, Amanda Bell

**Affiliations:** 1 Distributed Learning and Rural Initiatives, Cumming School of Medicine, University of Calgary, Calgary, Alberta, T2N 4Z6, Canada; 2 Professional Development and Educational Scholarship, Queen's University, Faculty of Health Sciences, Kingston, Ontario, K7L 0E9, Canada; 3 Distributed Medical Education, Queen's University, School of Medicine, Kingston, Ontario, K7L 0E9, Canada; 4 Continuing Education and Professional Development, Northern Ontario School of Medicine, Thunder Bay, Ontario, P7B 5E1, Canada; 5 Distributed Medical Education, College of Medicine, University of Saskatchewan, Prince Albert, Saskatchewan, S6V 5T4, Canada; 6 Office of Rural and Regional Health, Faculty of Medicine and Dentistry, University of Alberta, Edmonton, Alberta, T6G 1C9, Canada; 7 Faculté de médecine, Université de Montréal, Trois-Rivières, Québec, G8Z 4E3, Canada; 8 Distributed Medical Education, Faculty of Medicine, Memorial University, St. John’s, Newfoundland, A1B 3V6, Canada; 9 Niagara Regional Campus, Michael G. DeGroote School of Medicine, McMaster University, St. Catharines, Ontario, L2S 3A1, Canada

**Keywords:** Distributed Medical Education, Faculty Engagement, Preceptor Recognition, Medical Education, Rural Medical Education

## Abstract

**Background**: Medical schools spend considerable time, effort, and money on recognition initiatives for rural and distributed medical education (DME) faculty. Previous literature has focused on intrinsic motivation to teach and there is little in the literature to guide institutional recognition efforts or to predict which items or types of recognition will be most appreciated.

**Methods:** To better understand how rural and DME faculty in Canada value different forms of recognition, we asked faculty members from all Canadian medical schools to complete a bilingual, national online survey evaluating their perceptions of currently offered rewards and recognition. The survey received a robust response in both English and French, across nine Canadian provinces and one territory.

**Results:** Our results indicated that there were three distinct ways that preceptors looked at recognition; these perspectives were consistent across geographic and demographic variables. These “clusters” or “currencies of recognition” included: i) Formal institutional recognition, ii) connections, growth and development, and iii) tokens of gratitude. Financial recognition was also found to be important but separate from the three clusters. Some preceptors did value support of intrinsic motivation most important, and for others extrinsic motivators, or a mix of both was most valued.

**Conclusions:** Study results will help medical schools make effective choices in efforts to find impactful ways to recognize rural and DME faculty.

## Introduction

Physician involvement in medical education is essential for the training of medical students and residents. This requires practicing physicians to take on additional roles as preceptors and teachers. In addition to their clinical supervision and teaching responsibilities, preceptors guide and mentor medical learners as they navigate the transition from academic settings to the realities of clinical practice. Preceptors provide professional orientation, socialization, and personal support for their medical learners ^1^.

Despite the vital role that preceptors play in medical education, institutions including medical schools often have difficulty in recruiting and retaining physicians who are willing to teach. Similar issues have been reported in the disciplines of nursing and pharmacy ^2, 3^. Mercer (2018) concluded that there is a shortage of family medicine physicians willing to supervise clerkships and electives. Multiple reasons for this shortage were identified, including increasing medical school enrollment, physician burnout, and disruptions in providing clinical care to patients ^4^.

Although it is widely acknowledged that preceptors are important resources, the medical education literature provides little information on the key issues of recruiting, recognizing, rewarding, and retaining them. Furthermore, there is a major knowledge gap about engaging faculty preceptors who work outside of academic health science centres in rural or remote practice or in distributed medical education (DME) settings. Engagement efforts, rewards, and incentives directed toward DME faculty vary across institutions. Unfortunately, there is little to guide institutions in selecting or prioritizing these well-intentioned recognition efforts.

Pink’s work on human motivation describes intrinsic motivators (mastery, purpose, and autonomy) and extrinsic motivators (salary, recognition, and reward), and posits that intrinsic motivators are inherently more motivating ^5^. Prior research has emphasized the importance of intrinsic motivators to teach over extrinsic motivators and suggested that engagement efforts focus on supporting intrinsic motivation ^6, 7^. Zelek and Goertzen (2018) connected Pink’s work to the engagement of DME faculty. They suggested that support of the intrinsic motivations of DME faculty was key and that a better overall understanding of both intrinsic and extrinsic factors relating to DME faculty engagement was required ^8^. Other literature has also supported the importance of extrinsic motivators. A study examining willingness to teach among general practitioners in Germany found that engagement was related to intrinsic interest in teaching, but that fair compensation was also important ^9^. A study of community preceptors in North Carolina found that monetary compensation was an important extrinsic motivator ^10^.

This study explores the diverse types of recognition that are provided to teachers and preceptors in distributed medical education (DME). The term ‘currencies of recognition’ was chosen to highlight the idea that there are many possible expressions of recognition, and that these expressions may hold different value to preceptors. Such initiatives may be associated with varying costs to the institution providing them. For example, supporting extrinsic motivation through providing financial remuneration to preceptors may be limited in an era of constrained budgets and cost-saving measures. In fact, such compensation may not be as highly valued by preceptors as opportunities to support their intrinsic motivation. For example, preceptors may prefer an environment where they are given recognition by their peers and students; this can be achieved at little to no financial cost to the institution.

Through surveys with DME faculty across Canada, we systematically explored what forms of recognition are most attractive, effective, and meaningful in engaging and rewarding community preceptors for their teaching work. The results of this project have important implications for preceptors: encouraging physicians to become new preceptors, as well as increasing satisfaction and retention of current preceptors. This study provides assistance to medical schools on a range of preceptor engagement strategies, for the effective support, remuneration, and retention of faculty in traditionally difficult-to-fill roles.

## Methods

### Ethics statement

The current study was reviewed and approved by the University of Calgary Conjoint Health Research Ethics Board (CCHREB) – Ethics ID REB19-1132. The survey was online and anonymous, and written informed consent was obtained from participants prior to proceeding to the survey. The survey was provided in both English and French. Participants could opt into providing contact information for participation in the qualitative phase two of the study. This contact information was collected in a separate database, not linked to survey responses. The CCHREB required that data be kept on a secure local server only for a period of seven years after completion of the study and then deleted.

### Study design

A national interest group centred on DME faculty engagement was formed at a DME meeting of the Association of Faculties of Medicine in Canada (AFMC) during the the Canadian Conference on Medical Education (CCME) in May 2019. Members of the group met every one to two months via teleconference or over Zoom throughout the study. The study group included a range of individuals involved with DME, including individuals in leadership roles, research roles and administrative roles. Individual members of the group were involved with DME in both urban and rural settings. The group included members from both French language and English language medical schools. All members of the group had previous research experience and specific members of the group had expertise in each of quantitative research, qualitative research, and statistics.

The research team created a survey tool, and a professional translator translated the final version into French so that it could be used across Canada. The survey ^11^ was piloted among DME leaders from across Canada in order to identify any additional potential forms of faculty engagement and recognition. The research group reviewed the pilot survey results and revised the survey to ensure that the breadth of faculty engagement and recognition efforts were captured. Changes made after the pilot survey ^11^ included adding detailed demographic questions, adding additional potential forms of recognition and separating questions about what forms of recognition are offered and what forms are meaningful. The survey collected data related to the current forms of recognition provided to DME preceptors, and the value preceptors place upon each recognition type.

Study recruitment was conducted through DME leaders at all Canadian medical schools. A bilingual introductory letter that included a link to the survey ^11^ on the Qualtrics survey platform ( http://www.qualtrics.com), was electronically emailed to DME leaders at each institution. The contacts at each school were the identified DME leads who make up the membership of the national AFMC-DME group. This cohort was requested to forward the letter and survey to their eligible DME faculty.

### Data collection and analysis

Survey data was collected between January 31 ^st^ and March 18 ^th^, 2020. Completed responses were downloaded from the Qualtrics survey platform. Numeric data were funneled into a Microsoft Excel spreadsheet. A member of the research team (NC) with quantitative expertise conducted statistical analyses using STATA version 16 ( https://www.stata.com/). Descriptive statistical techniques, such as means, percentages, and cross-tabulations, were used to describe respondents’ demographic characteristics and measures of meaningful and valuable forms of faculty recognition.

An exploratory factor analysis (EFA) – which aims to identify unobserved factors that explain the variation in sets of observed variables – was used to explore what distributed medical faculty considered to be important recognition at their institution ^12, 13^. The principal factors method via an orthogonal varimax rotation was used to extract a unidimensional construct called ‘important recognition’ ^14, 15^. This unidimensional construct produced high factor loadings ranging from 0.73 to 0.82 and Eigen values greater than 1.00 based on 13 items measuring the importance of faculty recognition, and was found to be highly internally consistent (α = 0.95). A conventional Kaiser-Meyer-Olkin (KMO) test value > 0.500 confirmed a sampling adequacy of the EFA ^13, 16^. Analysis of variance (ANOVA) tests were also conducted to determine if there were significant differences in these constructs across respondents’ demographic characteristics ^17^.

In addition to the numeric data, many respondents wrote in the free text fields provided on the survey. The written comments were exported from the survey instrument into MS Word tables and identifying information removed. Comments originally in French were translated into English by a professional translator in the Linguistics department at the University of Calgary. The data were then analyzed qualitatively in NVIVO 12 using a structured, inductive approach based on thematic analysis ^18^. Two members of the research team with qualitative expertise (RM and AB) performed independent analyses. Comments with multiple concepts could be assigned to more than one code; a process of constant comparison between codes was used to systematically categorize, compare, and evaluate the data. In order to ensure the trustworthiness and credibility of the analysis, after the first iteration of coding, RM and AB assessed whether they were achieving consensus with the coding. Thereafter, they met regularly to discuss memos, additional codes, and emerging themes. 

## Results

The survey yielded 226 usable responses. Responses were recorded from nine provinces (all except Prince Edward Island, which is a small province that does not have a medical school) and from the Northwest Territories. 22 respondents were from Quebec.

Respondents were asked to identify which forms of recognition were offered by their academic institutions and to rate how meaningful each form of recognition was to them personally. An exploratory factors analysis (EFA) was conducted as per the methods. Of the 22 items analyzed, the EFA results provided a distinct three-factor grouping. These factor groupings were highly internally consistent with a Cronbach alpha of 0.92 and included: formal institutional recognition (α = 0.89), connections, growth, and development (α = 0.88), and tokens of gratitude (α = 0.82). From the three distinct patterns of response, we developed the concept of ‘clusters of Recognition’ along with financial recognition, which was highly valued but separate from these three domains. ANOVA testing did not show any significant differences in these constructs or clusters of meaningful recognition across respondents’ demographic characteristics

**Figure 1.**
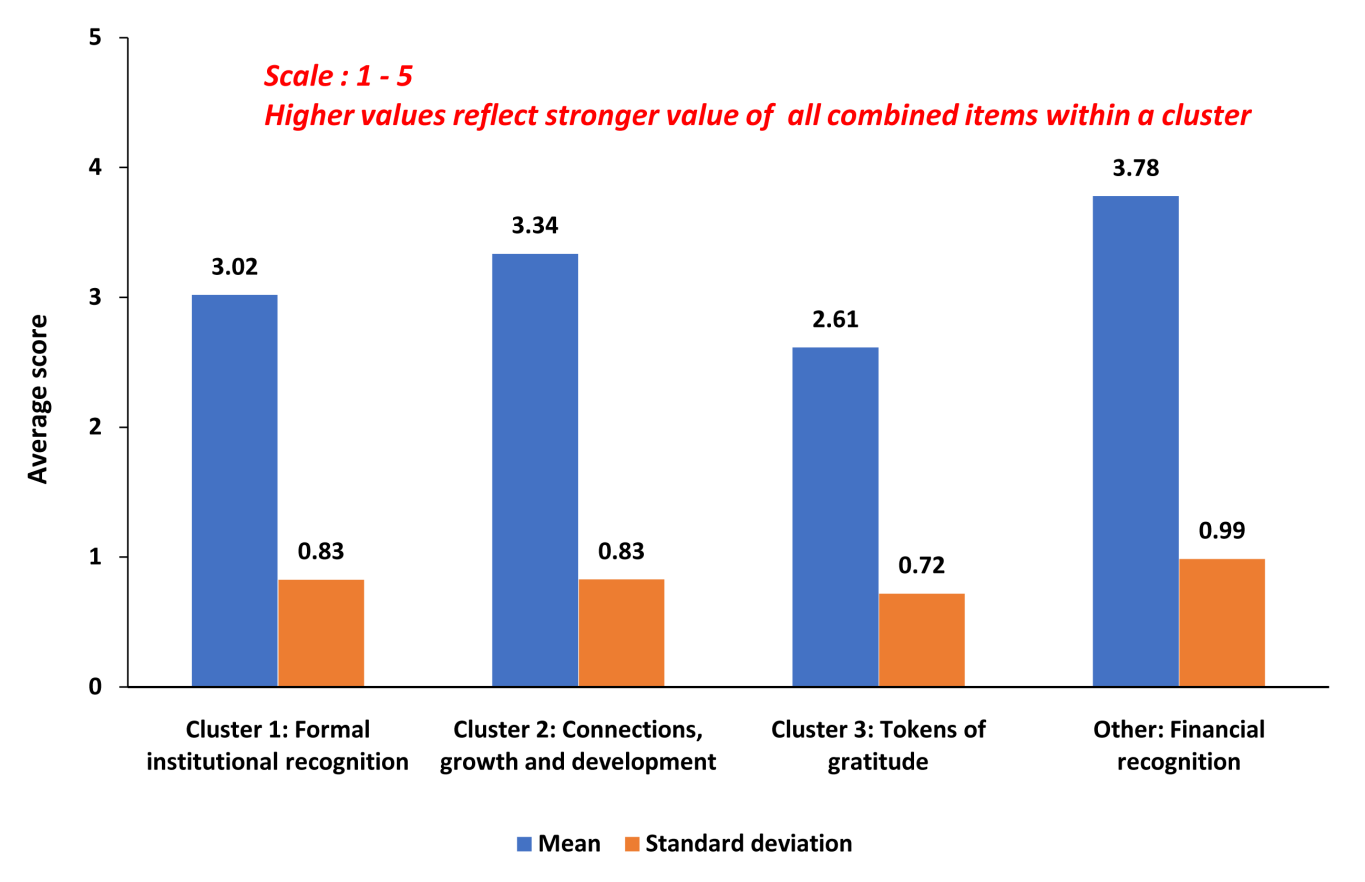
Clusters of statistically associated factors constituting meaningful faculty recognition.

Table 1 summarizes the clusters of recognition and the forms of recognition that are included in each. Some items are included in more than one cluster. Each item is shown along with its mean perceived value as rated on a five-point scale (x̅) and the frequency that DME faculty reported that it was offered by their academic institutions (P). Figure 1 shows the combined mean perceived value for all combined items within each cluster.

**Table 1.** Clusters of recognition and forms of recognition in each cluster.

Formal institutional recognition	Connections, growth and development	Tokens of gratitude	Financial recognition
DME specific awards (x̅ =3.31; *P* = 94.18%)	CME opportunities (x̅ =3.70; *P* = 97.95%)	Personal thank-you’s (x̅ =3.34; *P* = 94.30%)	Honoraria/Financial Remuneration (x̅ =3.77; *P* = 98.5%)
Academic promotion (x̅= 3.22; *P* = 93.26%)	Library access (x̅ =3.63; *P* = 92.23%)	Length of service recognition (x̅ =3.22; *P* = 93.78%)	
Institutional awards (x̅ =3.19; *P* = 94.44%)	Faculty development opportunities (x̅ =3.63; *P* = 96.45%)	Institution affiliation promotion (e.g. wall plaques (x̅ =2.77; *P* = 86.15%)	
Support for scholarship/research (x̅ =3.14; *P* = 93.26% )	Mentorship opportunities (x̅ =3.28; *P* = 96.88%)	Success stories publicized in newsletters (x̅ =2.75; *P* = 88.14%)	
Support for academic promotion (x̅ =3.13; *P* = 91.67%)	Networking opportunities (3.17; *P* = 91.79%)	Thank-you cards (x̅ =2.72; *P* = 87.11%)	
Recognition events (x̅ =2.89; )	Support for scholarship/research (x̅ =3.14; *P* = 90.05%)	Institution branded merchandise “swag” (x̅ = 2.30; *P* =73.7 %)	
Success stories publicized in newsletters (x̅ =2.75 *P* = 88.14%)	Support for academic promotion (x̅ =3.13; *P* = 91.67%)	Small gifts (2.04; *P* = 68.95%)	
Individual recognition in campus newsletters (x̅ = 2.51; *P* = 89.39%)	Site visits from institutional leadership (x̅ =3.01; *P* = 87.82%)	Holiday cards (x̅ =1.93; *P* =58.85%)	

Our qualitative analysis of free text from the survey supported the main findings of the quantitative analysis. Responses highlighted intrinsic motivations for teaching, including their identity as a teacher and wanting to give back through teaching. Comments around financial considerations focused on the substantial amount of unpaid work associated with teaching and the desire for fairness or parity with colleagues in academic centres. Participants highlighted the desire for meaningful connections with academic centres, as well as the feeling of disconnect caused by form letters and impersonal communication. Preceptors emphasized that connection with the students themselves and recognition by students was particularly meaningful.

In conjunction with the quantitative questions about the types of preceptor recognition that were offered by institutions and personally valued, DME faculty were also asked to elaborate about these rewards in four free text fields. 52 participants (23%) answered the question, “What other recognition does your institution currently offer DME faculty?” For the question “What recognition is NOT offered but would be beneficial?” there were 52 responses (23%). 24 participants (11%) completed the question “What other forms of recognition would you find personally meaningful?” The question “What does being a preceptor mean to you?” was answered by 155 participants (69%). Two other comment fields were included in the survey: a question asking for comments specifically about preceptor recognition was answered by 74 DME faculty (33%) and a question about general comments garnered 21 responses (9%). 51 of 378 individual free text responses were recorded in the French language. 

Our qualitative analysis of free text from the survey supported the main findings of the quantitative analysis, including the ‘clusters’ of recognition found in the EFA. Survey respondents agreed that it was vital for institutions to recognize and reward DME faculty for their contributions. Preceptors noted that many of their institutions did provide formal recognition, such as academic titles (e.g., ‘Clinical lecturer’), awards given out at gala events, or certificates for teaching. However, there was also a sense that rural preceptors were often “ignored or forgotten” (P171). For example, the award nomination process could disadvantage distributed faculty: “Many awards do not focus on clinical skills which precludes rural practitioners, who have less of an opportunity to do research and teach frequently. I find it very hard to nominate my colleagues, who are very deserving, for the available awards, and would like to be able to do so” (P182).

Institutions also provided DME faculty with various tokens of appreciation, such as generic thank-you cards, small gifts (“swag”) or institution-branded merchandise. Although such tokens account for a substantial portion of DME department budgets, many preceptors did not find these items to be meaningful. As one preceptor noted, “[It] would be nice if there was a bit more personalized effort to say ‘Thank you’ time to time” (P18).

Comments from the respondents also indicated the high value placed on connections and development. DME preceptors appreciated being affiliated with their institutions and networking with fellow preceptors. They emphasized the importance of the relationship with learners and noted that recognition by students was particularly meaningful. Continuing Medical Education (CME) training and Faculty Development courses were also valued. However, some preceptors stated that offerings could be inconvenient to attend: “…A lot available online but not generally at times conducive to actually working as a physician” (P159).

Financial compensation was a recurring theme in the comments. Preceptors acknowledged that they were paid a stipend/honorarium for teaching. They also mentioned that much of their motivation for teaching was intrinsic. However, respondents noted that taking on learners is inherently stressful and a substantial amount of their precepting workload is not compensated: “Being paid adequately would be nice. Teaching is actually the hardest part of my job. It's psychologically demanding and time consuming” (P33). Preceptors often reported concerns about the “fairness” of compensation compared to their colleagues in larger centers. For example, one DME faculty member said, “…We have been paid the same stipend for 28 years. We've never had an increase, despite the fact that teaching does take up a lot of time. No raise in 28 years. Pretty sure my urban counterparts have had raises, or maybe other perks” (P145). Some preceptors also pointed out that low remuneration makes it difficult to recruit other clinical teachers: “Preceptors need to be adequately compensated financially. It takes time to teach and if as a family physician your income is based on fee for service. Appropriate reimbursement will attract more preceptors” (P193).

Although financial compensation for teaching is generally viewed as inadequate, and preceptors often felt undervalued, most of them did not simply request more money. Survey respondents made several suggestions for low-cost forms of recognition that would be meaningful for them. Suggestions included written notes, regular site visits by leadership, DME-specific awards, library access, milestone recognition, and being recognized by peers/learners.

In addition to the survey questions about what recognition is currently provided and what recognition would be meaningful, we also asked “What does being a preceptor mean to you?” The participants’ answers to this question provide insight and context to their evaluation of current recognition. For example, several DME faculty mentioned that they had received help, support, or mentorship from others during their career. Becoming a preceptor allowed them to provide those services to others: “Gives me a chance to give back to the medical community in the same way that I was educated. To pass the torch to the next generation and provide mentorship is very valuable” (P110). Responses also highlighted intrinsic motivations for teaching, including the participant’s identity as a teacher. Most preceptors enjoyed teaching and believed that supervising learners kept their own clinical skills current.

## Discussion

Recognition of DME teaching faculty is an important component of faculty engagement, which impacts both recruitment and retention. Previous work on the engagement of rural and distributed faculty has emphasized the importance of intrinsic factors in the motivation of faculty to teach ^6– 8^. Although academic institutions devote time and resources to extrinsic recognition of their rural and distributed faculty, there is limited literature to guide these efforts ^9, 10^. As a result, there is a risk that these initiatives might be ineffective or misperceived.

The results of our study indicate that rural and distributed teaching faculty have three distinct perspectives about recognition efforts, which we termed ‘clusters of recognition’. We named each cluster based on the items highlighted as important among respondents in each group: i) formal institutional recognition, ii) connections, growth, and development, iii) tokens of gratitude. Financial recognition was also found to be highly valued but was separate from the three clusters.

Both the quantitative analysis and the free text comments confirmed the importance of the intrinsic motivation to teach. The ‘connections, growth, and development’ cluster of recognition showed preference for items that support intrinsic motivation such as continuing medical education (CME) opportunities, library access, faculty development, and mentorship.

Extrinsic motivators were also found to be an important motivator among DME faculty in our study. The ‘tokens of gratitude’ cluster of recognition also centered around extrinsic motivators such as personal thank-you’s, length of service recognition, and promotion of institutional affiliation. Some survey respondents showed preference for both intrinsic and extrinsic motivators. The ‘formal institutional recognition’ cluster contains both items that support intrinsic motivation, such as support for research and scholarship, and items that support extrinsic motivation such as DME specific awards.

Financial compensation and financial fairness and equity was highly valued and separate from the other clusters of recognition. Free text comments around financial remuneration indicated that most preceptors did not ask for more money; instead, they were concerned with parity of financial remuneration with urban peers, fairness, and recognition of the large amount of unpaid work associated with teaching. Fair financial compensation seems to be an important extrinsic motivator for DME faculty and is unconnected to preferences in the other clusters of recognition.

Overall, these results identify three ‘currencies of recognition’ which are offered by academic institutions and are valued by their preceptors. Our study suggests that there is a diversity of viewpoints among DME faculty about engagement and recognition. Some preceptors highlighted the importance of intrinsic motivation and having it supported, while other DME faculty stressed the importance of forms of extrinsic motivation. Although it is unlikely any one institution can include all forms of recognition, targeted efforts can be made to include at least some items from each cluster in order to meet the differing needs of their faculty and to purposefully support both intrinsic and extrinsic motivations to teach. Interestingly, ‘swag’ - small gifts and institutionally branded merchandise, which are common and expensive forms of recognition provided by academic rural and DME offices, were among the lowest scored items in terms of value.

Despite our broad Canadian sample of rural and distributed faculty, it is possible that there are additional clusters of recognition or faculty perspectives that were not captured; we recognize this is a potential limitation of our study.

## Conclusion

Our study included a national sample of rural and distributed faculty including both English and French respondents. We identified three important clusters or perspectives of faculty recognition as well as important contextual information around financial recognition. Our results demonstrate that DME faculty have varied viewpoints regarding recognition and engagement. Both intrinsic and extrinsic motivators were found to be important. Fairness around financial recognition was of particular concern. The results of this study can be used by medical schools and academic rural and distributed medical education groups to choose a variety of forms of faculty recognition, both intrinsic and extrinsic, that will meet the varied needs of DME teaching faculty. A follow-up study has recently been completed and results will be published shortly. This second study used qualitative methodology to further explore the motivations among DME faculty to teach and what forms of recognition are most important to them.

## Data availability

### Underlying data

The underlying data for this study includes potentially identifying information as responses included free text and geographic responses from areas with very few possible study subjects. The data is held on a secure server and only accessible by the research team as described in the data handling requirements approved by the University of Calgary Conjoint Health Research Ethics Board for this study (Ethics ID REB19-1132). The CCHREB approval for this study requires that the data be held on a secure local server for a period of seven years beyond the completion of the study. Research or medical education groups conducting related studies with specific questions relating to the data can contact the corresponding author (Dr. Johnston) to determine if de-identified aggregate data relating to the question can be shared.

### Extended data

Open Science Framework: Currencies of recognition: What rewards and recognition do Canadian distributed medical education preceptors value?

https://doi.org/10.17605/OSF.IO/W9HG2 ^11^.

This project contains the following extended data:

- DME Recognition Pilot survey (Survey testing prior to the study in English as the pilot language).
- DME Recognition survey (Final survey used in the study in French and English).

Data are available under the terms of the Creative Commons Zero “No rights reserved” data waiver (CC0 1.0 Public domain dedication).

### Reporting guidelines

Figshare: SRQR checklist for ‘Currencies of recognition: What rewards and recognition do Canadian distributed medical education preceptors value?’ https://doi.org/10.17605/OSF.IO/W9HG2 ^11^.

Data are available under the terms of the Creative Commons Zero "No rights reserved" data waiver (CC0 1.0 Public domain dedication).
