# Supplementary material for: Currencies of recognition: What rewards and recognition do Canadian distributed medical education preceptors value?
Source: MedEdPublish (2016). 2022 Mar 1;12:12. [Version 1] doi: 10.12688/mep.17540.1 (PMC9370089; doi:10.12688/mep.17540.1)
Supplement: Peer review history

# Peer review: Reviewer response for version 1

**DOI:** 10.21956/mep.18815.r31699

**Published:** 2022-03-18

**Authors:** Randall Longenecker

**Affiliations:** 1 The RTT Collaborative, Athens, OH, USA

This article addresses both intrinsic and extrinsic rewards of precepting and ways of acknowledging the value of community faculty. It is very well written, and the conclusions are well supported. I look forward to a follow-up qualitative study directly engaging with the distributed medical education and training community. Both studies represent an important contribution to the literature giving seldom heard voice to the perspective of community preceptors.

 I would have liked to have seen more demographics, demonstrating the ‘distributed’ nature of this sample, e.g., rurality, age, years of precepting, and specialty or discipline, even if any one sub-group was too small for quantitative analysis. I also think the information about associated factors constituting meaningful faculty recognition would have better been presented in a graph of confidence intervals for each form of recognition, with bars and whiskers. I suspect the variety of responses with 95% confidence intervals may have better made the point of individual variation. Chapman and White describe five languages of appreciation in the workplace in their book by that title and make the point for individualizing recognition (Chapman & White (2011 ^1^)).

 This work resonates with and validates my own experience as a distributed rural preceptor. Most important is the conclusion: “Our results demonstrate that DME faculty have varied viewpoints regarding recognition and engagement. Both intrinsic and extrinsic motivators were found to be important...The results of this study can be used by medical schools and academic rural and distributed medical education groups to choose a variety of forms of faculty recognition, both intrinsic and extrinsic, that will meet the varied needs of DME teaching faculty.” One size does not fit all!

Have any limitations of the research been acknowledged?

Yes

Is the work clearly and accurately presented and does it cite the current literature?

Yes

If applicable, is the statistical analysis and its interpretation appropriate?

I cannot comment. A qualified statistician is required.

Are all the source data underlying the results available to ensure full reproducibility?

Yes

Is the study design appropriate and is the work technically sound?

Yes

Are the conclusions drawn adequately supported by the results?

Yes

Are sufficient details of methods and analysis provided to allow replication by others?

Yes

Reviewer Expertise:

Rural medical education and training

I confirm that I have read this submission and believe that I have an appropriate level of expertise to confirm that it is of an acceptable scientific standard.

## References

[1] : Five Languages of Appreciation in the Workplace The.2011;

# Peer review: Reviewer response for version 1

**DOI:** 10.21956/mep.18815.r31700

**Published:** 2022-03-07

**Authors:** Lisa Graves

**Affiliations:** 1 Department of Family and Community Medicine, Western Michigan University Homer Stryker M.D. School of Medicine, Kalamazoo, MI, USA

This is a well written paper exploring an important aspect of distributed medical education.  The abstract was well written, but could benefit from a stronger conclusion as it appears that the authors have made a strong case for their hypotheses.

- The Introduction sets the stage well.
  The Methods section would benefit from a further explanation of study participant recruitment and how bias was avoided.
- Results were well documented.  A response rate would be appreciated.  An explanation for an international audience of the importance of the Quebec respondents would be helpful.  Demographic data such as age , gender, specialty were not reported.  It would be appropriate to include this information or describe why this was not done.  Reporting of std deviation in figure is non traditional and may confuse.  
- The discussion was well written and supported.  The issue of parity is critical and consideration should be given to further highlighting this. 

Overall, an informative paper.

Is the work clearly and accurately presented and does it cite the current literature?

Yes

If applicable, is the statistical analysis and its interpretation appropriate?

Partly

Are all the source data underlying the results available to ensure full reproducibility?

Yes

Is the study design appropriate and is the work technically sound?

Yes

Are the conclusions drawn adequately supported by the results?

Yes

Are sufficient details of methods and analysis provided to allow replication by others?

Yes

Reviewer Expertise:

medical education

I confirm that I have read this submission and believe that I have an appropriate level of expertise to confirm that it is of an acceptable scientific standard.
